# Distinct inflammatory profile underlies pathological increases in creatinine levels associated with *Plasmodium vivax* malaria clinical severity

**DOI:** 10.1371/journal.pntd.0006306

**Published:** 2018-03-29

**Authors:** Luís A. B. Cruz, Manoel Barral-Netto, Bruno B. Andrade

**Affiliations:** 1 Instituto Gonçalo Moniz, Fundação Oswaldo Cruz (FIOCRUZ), Salvador, Brazil; 2 Curso de Medicina, Faculdade de Tecnologia e Ciências, Salvador, Brazil; 3 Multinational Organization Network Sponsoring Translational and Epidemiological Research (MONSTER) Initiative, Fundação José Silveira, Salvador, Brazil; 4 Universidade Federal da Bahia, Faculdade de Medicina, Salvador, Brazil; 5 Instituto Nacional de Ciência e Tecnologia, Instituto de Investigação em Imunologia, São Paulo, Brazil; 6 Escola Bahiana de Medicina e Saúde Pública, Salvador, Brazil; 7 Universidade Salvador (UNIFACS), Laureate Universities, Salvador, Brazil; Temple University, UNITED STATES

## Abstract

**Background:**

Although *Plasmodium vivax* infection is a frequent cause of malaria worldwide, severe presentations have been more regularly described only in recent years. In this setting, despite clinical descriptions of multi-organ involvement, data associating it with kidney dysfunction are relatively scarce. Here, renal dysfunction is retrospectively analyzed in a large cohort of vivax malaria patients with an attempt to dissect its association with disease severity and mortality, and to determine the role of inflammation in its progression.

**Methods:**

A retrospective analysis of a databank containing 572 individuals from the Brazilian Amazon, including 179 patients with *P*. *vivax* monoinfection (161 symptomatic malaria, 12 severe non-lethal malaria, and 6 severe lethal disease) and 165 healthy controls, was performed. Data on levels of cytokines, chemokines, C-reactive protein (CRP), fibrinogen, creatinine, hepatic enzymes, bilirubin levels, free heme, and haptoglobin were analyzed to depict and compare profiles from patients per creatinine levels.

**Results:**

Elevated creatinine levels were found predominantly in women. Vivax malaria severity was highly associated with abnormal creatinine increases, and nonsurvivors presented the highest values of serum creatinine. Indication of kidney dysfunction was not associated with parasitemia levels. IFN-γ/IL-10 ratio and CRP values marked the immune biosignature of vivax malaria patients, and could distinguish subjects with elevated creatinine levels who did not survive from those who did. Patients with elevated serum creatinine or severe vivax malaria displayed indication of cholestasis. Biomarkers of hemolysis did not follow increases in serum creatinine.

**Conclusion:**

These findings reinforce the hypothesis that renal dysfunction is a key component in *P*. *vivax* malaria associated with clinical severity and mortality, possibly through intense inflammation and immune imbalance. Our study argues for systematic evaluation of kidney function as part of the clinical assessment in vivax malaria patients, and warrants additional studies in experimental models for further mechanism investigations.

## Author summary

Severe clinical presentations of *Plasmodium vivax* malaria are not completely understood. Multi-organ involvement is described in severe vivax cases, however data associating it with kidney dysfunction are relatively scarce, in part because the clinical signs only appear late during kidney injury. We analyzed biomarkers of renal function in groups of patients from the Brazilian Amazon with different presentations of vivax malaria to determine its associations with disease progression. Inflammatory biomarkers were also analyzed to assess inflammation related to kidney dysfunction. The results indicate that severe disease presentation in these patients was associated with abnormal serum creatinine elevations and exacerbated systemic inflammatory response. The highest levels of creatinine were observed in nonsurvivors. Biomarkers of hemolysis did not directly follow increases in serum creatinine. These readouts suggest that kidney dysfunction probably influences vivax malaria severity and mortality. As *P*. *vivax* is a widely distributed species of *Plasmodium* in the world, and severe cases are increasingly being reported, it is important to better understand the role of kidney injury in these presentations, especially considering that it may affect clinical outcomes.

## Introduction

Malaria is an infectious disease known for millennia. Although substantial investments have been made in the last couple of decades, with slight improvements regarding control of disease transmission in some endemic territories, the disease is still responsible for over 200 million cases annually, with almost 500,000 deaths only in 2015, especially among children [1]. *Plasmodium vivax* has a wide geographical distribution and was historically associated with milder disease presentations, while *Plasmodium falciparum* has been commonly related with increased severity and mortality [1–3]. Nevertheless, severe cases caused by *Plasmodium vivax* monoinfection have been frequently reported in recent years [1,4] and increasing interest resulted in several studies focused on the details of the vivax malaria clinical characteristics and pathogenesis [5–8].

Severe *P*. *vivax* cases have been associated with impaired immune response and inflammation-driven hepatic damage [5,9], as well as kidney involvement, among other symptoms [5,10–14]. While the mechanisms driving inflammatory damage in some key organs such as liver are quite well described in vivax malaria patients [4–6,9], scarce data are available with regard to the mechanisms of kidney injury during acute and severe disease [10]. In individuals with *P*. *falciparum* infection, acute kidney injury (AKI) has been linked to acute tubular necrosis (ATN) [4,15], associated with altered cytoadhesion of infected erythrocytes [16–20]. Whether these mechanisms are common in *P*. *vivax*-related AKI or whether there are unique features that distinguishes the kidney damage caused by infection with different *Plasmodium* species is unknown.

In some reports, vivax malaria patients have been described to exhibit oliguria or anuria [10–13], and in those cases, hemodialysis treatment has been employed as the kidney failure had already established. The use of a widely available and easily measured laboratory parameter that could faithfully readout kidney damage during acute malaria has potential to optimize clinical management. In the present study, the relevance of serum creatinine levels in evaluation of patients with *P*. *vivax* malaria was tested and associated with the systemic inflammatory profile and clinical outcomes. These analyzes were aimed to examine how much of the parasite-host relationships, and especially systemic inflammation, would play a role in the settlement of vivax malaria-associated AKI.

## Methods

### Ethics statement

Written informed consent was obtained from all participants or their legally responsible guardians, and all clinical investigations were conducted according to the principles expressed in the Declaration of Helsinki. The project was approved by the institutional review board of the Faculdade de Medicina, Faculdade São Lucas, Rondônia, Brazil, where the study was performed.

### Study design

The present study is a retrospective assessment of a databank containing epidemiological, immunological, and clinical data from 572 subjects recruited between 2006 and 2007 from the Brazilian Amazon (Rondônia, Brazil), as a part of a finalized project that described determinants of susceptibility to vivax malaria and from which several investigations have been already reported [5,9,21–28]. The details of the recruitment, diagnosis and clinical definitions were published previously [5,21–27]. The primary investigations included passive case detection, from individuals who sought care at the municipal hospital in Buritis (Rondônia, Brazil) or at Brazilian National Foundation of Health (FUNASA) diagnostic centers. In addition, active case detection was performed and included home visits with interviews, clinical evaluations and specimens sampling for laboratory assays in the municipalities of Buritis and Demarcação (Rondônia, Brazil). Blood collection for nested polymerase chain reaction (PCR) and other measurements (cytokines, chemokynes, hepatic enzymes, creatinine, fibrinogen, bilirubin levels, free heme and haptoglobin) were performed at the time of study enrollment, meaning that specimens were collected at diagnosis, during acute phase of disease in malaria patients, before treatment initiation. For the present study, only patients with *P*. *vivax* monoinfection (n = 179) and healthy controls (n = 165, from which 152 had all the epidemiological data available) were included. The exclusion criteria for the present study were: asymptomatic *P*. *vivax* monoinfection, documented *P*. *falciparum* or HIV infections, tuberculosis, cancer, or use of immunosuppressant drugs. Noteworthy, as there were no consensus to define severe vivax malaria, we adapted the criteria used to define severe disease caused by *P*. *falciparum* infection, as published previously by our group [5]. Clinical, demographic and epidemiological characteristics of the participants included in the current study are described in Table 1, S1 Table and S2 Table.

**Table 1:** Characteristics of the *Plasmodium vivax* malaria patients with elevated or normal creatinine levels.

Characteristic	Elevated Creatinine Levels	Normal Creatinine Levels	P-value
**N**	89	90	
**Women**–no. **(%)**	47 (52.80)	32 (35.55)	0.0242
**Age**–y **(IQR)**	34 (22–49)	32 (25.75–42)	0.5475
**Severe *vivax***–no. **(%)**	15 (16.85)	3 (3.33)	0.0027
**Hospitalized**–no **(%)**	14 (15.73)	1 (1.11)	0.0003
**Nonsurvivors**–no. **(%)**	6 (6.74)	0 (0)	0.0138
**Parasitemia**–count/μL **(IQR)**	6453 (1234–49844)	6324 (851–72985)	0.9055

Frequency data were compared using the exact Fisher’s test, while continuous variables (age and parasitemia) were compared using the Mann-Whitney *U* test. Subjects were classified as having elevated creatinine levels if serum creatinine values were above 1.24mg/dL for women, and 1.29mg/dL for men.

### Laboratory measurements

Data on several mediators were selected in order to assess the overall immune response; and all the biomarkers contained in the databank were analyzed. Plasma levels of cytokines IL-1β, IL-4, IL-6, IL-10, IL-12p70, IFN-γ, tumor necrosis factor (TNF)-α, and of chemokines CCL2, CCL5, CXCL9, and CXCL10 were measured using the Cytometric Bead Array—CBA (BD Biosciences Pharmingen, San Diego, CA, USA), according to the manufacturer’s protocol. Circulating concentrations of total heme in plasma samples were estimated using the QuantiChrom Heme Assay Kit (BioAssay Systems, Hayward, CA), according to the manufacturer’s protocol. The following procedure was performed to distinguish heme bound to hemoglobin *vs*. non-hemoglobin heme, i.e. free heme [27]. Plasma absorbance was measured in the UV-VIS range at λ = 576nm (peak of absorption for hemoglobin) and λ = 630nm using a using NanoDrop spectrophotometer (NanoDrop Technologies). The following formula: [hemoglobin-heme] = (66 x λ = 576nm)-(80 x λ = 630nm) was applied and referred to a calibration curve of purified hemoglobin ranging from 320–5 μM hemoglobin-heme equivalents. Free heme was calculated according to the following formula: [Free heme] = [Total heme]-[hemoglobin-heme]. The measurements of aspartate amino-transferase (AST), alanine amino-transferase (ALT), total bilirubin, direct bilirubin, creatinine, fibrinogen and C-reactive protein (CRP) were performed at the Pharmacy School of the Federal University of Bahia and at the clinical laboratory of Faculdade São Lucas.

### Stratification of patients based on serum creatinine and bilirubin levels

Serum creatinine levels from the 179 malaria vivax patients were considered as elevated or normal considering thresholds established by gender, as previously described [29]. Elevated creatinine levels were defined in female patients as values above 1.24mg/dL and in male individuals as higher than 1.29mg/dL [29]. Elevated total bilirubin levels were defined as higher than 1.5mg/dL whereas high indirect or direct bilirubin levels infer concentrations above 1.2mg/dL or 0.3mg/dL, respectively [30].

### Statistical analysis

The median values with interquartile ranges (IQR) were used as measures of central tendency. The Fisher’s exact test was used to compare frequencies between the study groups. Continuous variables were compared between the study groups using the Mann-Whitney *U* test (2-group comparisons), or the Kruskall-Wallis test with Dunn’s multiple comparisons ad hoc test (between 3 or more groups). Hierarchical cluster analyzes were performed using the Ward’s method with bootstrap (100X). A p-value below 0.05 was considered statistically significant after adjustments for multiple comparisons using the Holm’s Bonferroni test. The statistical analyzes were performed using Graphpad Prism 6.0 (GraphPad Software Inc., San Diego, CA, USA), and JMP 12.0 (SAS, Cary, NC, USA).

## Results

### Characteristics of the study participants

Among vivax malaria patients, those categorized as having elevated creatinine levels (n = 89) had similar age, but were more frequently female than individuals with normal levels (n = 90) (Table 1). Overall, patients with *P*. *vivax* malaria were younger than uninfected individuals (median age: 32yrs, IQR: 24–47 vs. 39yrs, IQR: 25–51, P = 0.0096), but were undistinguishable with regard to gender distribution (male frequency: 55.9% vs. 47.4%, P = 0.151). Patients with severe *P*. *vivax* infection recalled less previous malaria episodes and lived for shorter time in the endemic area than individuals with mild infection (S1 Table). Vivax malaria patients exhibiting elevated serum creatinine levels presented sweating, diarrhea, dehydration, abdominal pain, hepatomegaly, jaundice and disorientation more frequently than those with normal creatinine values (S2 Table).

### Creatinine levels in severe malaria vivax

**Fig 1:**
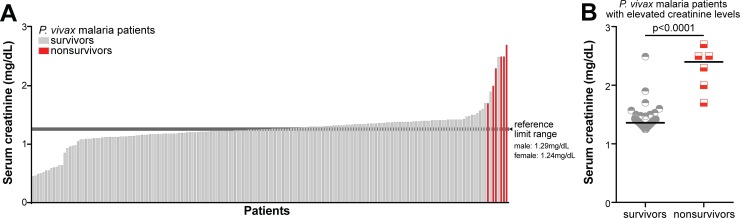
Distribution of serum creatinine levels and clinical outcomes in patients with acute *Plasmodium vivax* malaria. (A) Histogram representing serum creatinine levels of the 179 patients with *P*. *vivax* monoinfection; the patients are colored accordingly to the disease outcome. Survivors are colored in grey, while nonsurvivors are colored in red. Reference intervals for men and women are displayed by transversal lines, with lower limit representing its reference for women, and upper limit representing its reference for men. (B) Scatter-plot of the creatinine levels presented by the subgroups of nonsurvivors and survivors amongst the subjects with elevated serum creatinine. Data analysis was performed using the Mann-Whitney *U* test. Bars represent median values.

Among vivax malaria patients, the frequency of individuals presenting with severe disease was significantly higher in patients with elevated creatinine levels than in those who had normal values (16.85% vs. 3.33%, P = 0.0027, Table 1). Following a similar pattern, frequency of hospitalization was also elevated in patients with elevated creatinine levels vs. those with normal values (P = 0.0003, Table 1). Moreover, all patients who did not survive (n = 6) were among the group of individuals presenting with the highest creatinine levels (Fig 1A); two of them presented with anuric renal failure while the other four presented respiratory failure as the major presentation at admission (see also in [5]). Furthermore, within the group of patients with abnormally high creatinine values, nonsurvivors presented with even higher levels when compared with survivors (median: 2.4mg/dL, IQR: 1.925–2.55 vs. 1.36mg/dL, IQR: 1.31–1.41, P < 0.0001, Fig 1B).

### Elevated creatinine levels are associated with intense inflammatory imbalance in vivax malaria patients

**Fig 2:**
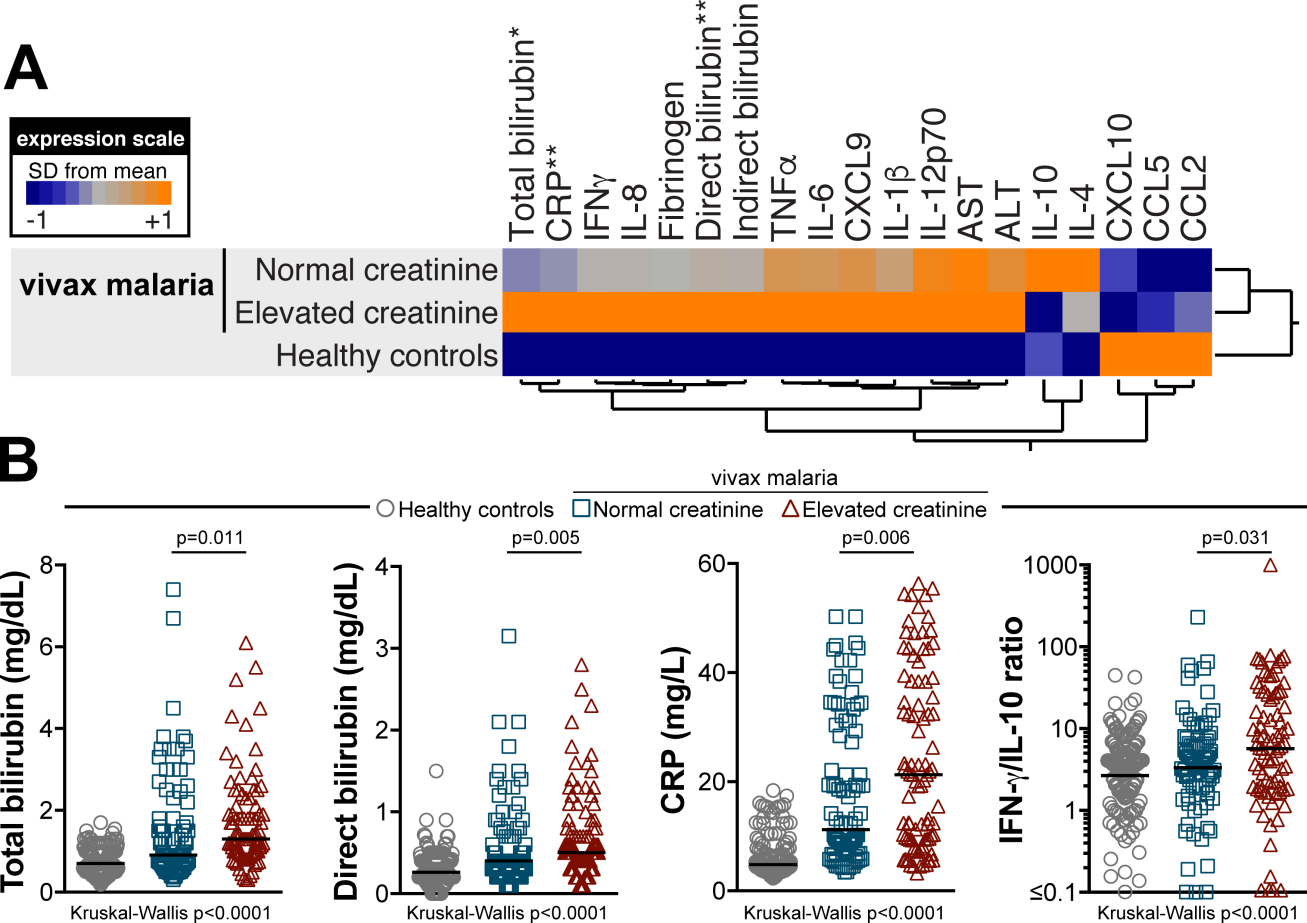
Inflammatory profile of patients with acute *Plasmodium vivax* malaria exhibiting abnormally high levels of creatinine. (A) Overall profile of plasma concentrations of several biochemical parameters, cytokines and chemokines in uninfected controls (n = 165) as well as in vivax malaria patients presenting with creatinine levels within the normal range (n = 90) or abnormally elevated (n = 89). Data were processed using hierarchical cluster analysis (Ward’s method) with 100X bootstrap. Dendograms represent hierarchical distance. Asterisks indicate parameters which were statistically different between the groups of malaria patients with normal or elevated creatinine levels assessed using the Mann-Whitney *U* test. Scatter plots of these parameters are shown in (B). The IFN-γ/IL-10 ratio, which has been shown to correlate with the inflammatory imbalance in vivax malaria (4), was also compared between the study groups. Bars represent median values.

Circulating levels of several cytokines, chemokines and inflammatory parameters were compared between the groups of *P*. *vivax* malaria patients presenting with or without elevated creatinine values. Patients who displayed elevated creatinine levels exhibited higher CRP values than that in those who did not (median: 21.30ng/mL, IQR: 9.45–38.45 vs. 11.20ng/mL, IQR: 7.125–29.58, respectively, P = 0.0057, Table 2 and Fig 2A and 2B). Neither IL-10 (median: 9.69pg/mL, IQR: 6.43–36.83 vs. 19.70pg/mL, IQR: 6.50–59.54, P = 0.0665) or IFN-γ (median: 132.0pg/mL, IQR: 35.0–333.5 vs. 85.48pg/mL, IQR: 40.15–321.5, P = 0.5326) levels were significantly different between the study groups (Table 2 and Fig 2A and 2B). Interestingly, values of IFN-γ/IL-10 ratio, which have been shown previously to correlate with systemic inflammation in vivax malaria [5], were increased in patients who had high creatinine levels compared with those who had not (median: 5.720 arbitrary units [AU], IQR: 1.675–25.21 vs. 3.334AU, IQR: 1.181–6.558, P = 0.0306, Table 2, Fig 2A and 2B).

**Table 2:** Biochemical evaluation of *Plasmodium vivax* malaria patients with elevated or normal creatinine levels.

Parameter	Elevated Creatinine Levels	Normal Creatinine Levels	P-value
**N**	89*	90*	
**TNF-α**– pg/mL	39.40 (20.75–83.80)	33.55 (12.55–78.85)	0.4221
**IFN-γ**– pg/mL	132.00 (35.00–333.50)	85.48 (40.15–321.5)	0.5326
**IL-1β**– pg/mL	11.40 (5.925–29.40)	10.09 (5.575–21.80)	0.2784
**IL-4**– pg/mL	28.47 (18.37–113.30)	31.25 (16.16–97.15)	0.6477
**IL-6**– pg/mL	69.20 (22.15–140.40)	59.10 (29.13–104.10)	0.5517
**IL-8**– pg/mL	32.50 (6.14–190.0)	20.54 (5.183–69.63)	0.1632
**IL-10**– pg/mL	9.69 (6.43–36.83)	19.70 (6.50–59.54)	0.0665
**IFN-γ/IL-10– AU**	5.720 (1.675–25.21)	3.334 (1.181–6.558)	**0.0306**
**IL-12p70**– pg/mL	20.94 (10.20–33.35)	20.45 (15.73–30.30)	0.8953
**CRP**–mg/L	21.30 (9.45–38.45)	11.20 (7.125–29.58)	**0.0057**
**CCL2 (MCP-1)**–ng/mL	73.10 (21.21–161.4)	61.24 (22.99–143.6)	0.7917
**CCL5 (RANTES)–**μg/mL	25739 (18530–79892)	24982 (14214–50209)	0.2061
**CXCL9 (MIG)**–ng/mL	2386 (507–11548)	2120 (368.1–6854)	0.2424
**CXCL10**– pg/mL	71.30 (26.07–445.8)	77.55 (25.17–273.9)	0.3810
**Fibrinogen**–mg/dL	400.2 (274.2–497.5)	309.5 (218.1–478.1)	0.0539
**AST**–U/L	167.4 (87.62–642.8)	166.3 (68.97–428.4)	0.3200
**ALT**–U/L	190.3 (133.7–495.2)	177.6 (112.6–352.2)	0.1842
**Total Bilirubin**–mg/dL	1.300 (0.965–2.100)	0.910 (0.687–1.725)	**0.0109**
**Elevated Total Bilirubin**–no. **(%)**	36 (40.45)	25 (27.78)	0.0841
**Elevated Total Bilirubin**–mg/dL	2.45 (1.825–3.150)	3.00 (2.25–3.60)	0.1562
**Direct Bilirubin-mg/dL**–mg/dL	0.500 (0.300–0.900)	0.400 (0.200–0.700)	**0.0054**
**Indirect Bilirubin-mg/dL**–mg/dL	0.800 (0.530–1.300)	0.625 (0.400–1.125)	0.0690
**Elevated Indirect Bilirubin**–mg/dL	1.5 (1.4–2.2)	2.3 (1.6–2.7)	**0.0353**
**Elevated Indirect Bilirubin**–no. **(%)**	24 (26.96)	19 (21.11)	0.3860
Free Heme*– μM	29.88 (15.67–44.82)	35.75 (25.24–55.06)	0.1332
Haptoglobin*–ng/mL	1470 (1128–2136)	1582 (1121–1810)	0.6063

Data represents interquartile range, except for count/frequency of patients with Elevated Indirect or Total bilirubin, shown in percentage for each respective column. Continuous variables were compared using the Mann-Whitney *U* test, while frequency data were compared using the exact Fisher’s test. Subjects were classified as having elevated creatinine levels if serum creatinine values were above 1.24mg/dL for women, and 1.29mg/dL for men; elevated total or indirect bilirubin levels were considered if values were higher than 1.5mg/dL or 1.2mg/dL respectively. AU = arbitrary unit.

*variables presented different number of patients from which data was available for: data of 29 and 28 patients presented elevated and normal creatinine levels respectively in the Free Heme– μM row, while 33 and 35 patients presented elevated and normal creatinine levels respectively in the Haptoglobin-–ng/mL row.

Circulating levels of all other cytokines, chemokines and acute phase proteins could not distinguish the two groups of patients stratified accordingly to serum creatinine levels, and are described in detail in Table 2.

Patients categorized as having elevated creatinine levels presented with higher total bilirubin concentrations than those who had normal creatinine values (median: 1.3mg/dL, IQR: 0.965–2.1 vs. 0.91mg/dL, IQR: 0.687–1.725, respectively, P = 0.0109, Table 2, Fig 2B). A similar pattern was observed with regard to direct bilirubin fraction (P = 0.0054, Table 2, Fig 2B), while no difference was found in indirect bilirubin levels between these study groups. Counterintuitively, when only patients with elevated indirect bilirubin levels (>1.2mg/dL) were analyzed (n = 43), higher values of this parameter were detected among the group of patients with normal serum creatinine (median: 2.33mg/dL, IQR: 1.6–2.7 vs. 1.53mg/dL, IQR: 1.4–2.2, P = 0.0453, Table 2). In addition, circulating concentrations of both free heme and haptoglobin were undistinguishable between the groups of patients presenting with high indirect bilirubin levels and elevated or normal creatinine values (Table 2). Furthermore, concentrations of hepatic aminotransferases were also similar between these subpopulations (Table 2).

Hierarchical cluster analysis of the z-score normalized circulating levels of the markers evaluated confirmed that individuals with elevated creatinine levels exhibited a distinct inflammatory profile compared to malaria patients with normal creatinine values or to uninfected healthy controls (S3 Table and Fig 2A).

### Associations between creatinine levels, inflammatory profile and mortality in *P*. *vivax* malaria

Additional analyses were performed to depict the overall systemic inflammatory profile between vivax malaria patients who survived and those who did not. Nonsurvivors presented higher CRP values than those who survived (median: 34.43ng/mL, IQR: 16.43–50.7 vs. 21.3ng/mL, IQR: 9.4–38.4 vs. 11.23ng/mL, IQR: 7.125–29.58, P = 0.0122, Table 3). In addition, values of IFN-γ/IL-10 ratio were increased among the nonsurvivors (median: 18.63AU, IQR: 8.29–31.55 vs. 5.075AU, IQR: 1.664–22.69 vs. 3.334AU, IQR: 1.181–6.558, P = 0.0282, Table 3). Circulating levels of all the other cytokines, chemokines and acute phase proteins could not distinguish these two groups that considered survival among patients with elevated serum creatinine, and are described in detail in Table 3. Hepatic aminotransferases and indirect bilirubin values were not significantly different as well. Nevertheless, nonsurvivors presented higher values of total bilirubin (median: 2.15mg/dL, IQR: 1.2–3.175 vs. 1.3mg/dL, IQR: 0.95–1.9 vs. 0.91mg/dL, IQR: 0.687–1.725, P = 0.0250, Table 3) and direct bilirubin (median: 1.1mg/dL, IQR: 0.325–1.675 vs. 0.5mg/dL, IQR: 0.3–0.8 vs. 0.4 mg/dL, IQR: 0.2–0.7, P = 0.0133, Table 3) when compared with the group of survivors. Moreover, when nonsurvivors were compared only with patients with normal creatinine levels, values of CRP (median: 34.4ng/mL, IQR: 16.43–50.7 vs. 11.20ng/mL, IQR: 7.125–29.58, respectively, P = 0.0121) and IFNγ/IL-10 ratio (median: 18.63AU, IQR: 8.29–31.55 vs. 3.334AU, IQR: 1.181–6.558, respectively, P = 0.0129) were increased in nonsurvivors whereas IL-12p70 concentrations were elevated in those with normal creatinine levels (median: 10.0pg/mL, IQR: 5.093–15.14 vs. 20.45pg/mL, IQR: 15.73–30.30, respectively, P = 0.0333)(Table 3).

**Table 3:** Biochemical evaluation of *Plasmodium vivax* malaria patients with regard to mortality.

Parameter	Elevated Creatinine Levels		Normal Creatinine Levels	P-value
	Survivors	Nonsurvivors		
**N**	83	6	90	
**TNF-α**– pg/mL	39.4 (20.1–84.2)	31.95 (18.93–76.8)	33.55 (12.55–78.85)	0.6774
**IFN-γ**– pg/mL	100 (32.4–342.1)	181.6 (62.25–357.2)	85.48 (40.15–321.5)	0.6522
**IL-1β**– pg/mL	11.4 (6.17–29.5)	15.54 (4.825–29.33)	10.09 (5.575–21.80)	0.5456
**IL-4**– pg/mL	27.95 (18.23–113.7)	36.42 (18.34–115.5)	31.25 (16.16–97.15)	0.8804
**IL-6**– pg/mL	61.2 (22.1–147.5)	101.2 (40.6–139.8)	59.10 (29.13–104.10)	0.6502
**IL-8**– pg/mL	31.33 (5.940–187.3)	66.84 (14.69–211.4)	20.54 (5.183–69.63)	0.2561
**IL-10**– pg/mL	9.54 (6.43–39.5)	11.5 (7.91–13.42)	19.70 (6.50–59.54)	0.1805
IFN-γ/IL-10*– AU	5.075 (1.664–22.69)	18.63 (8.29–31.55)	3.334 (1.181–6.558)	**0.0282**
IL-12p70*– pg/mL	21.30 (10.4–38.5)	10.0 (5.093–15.14)	20.45 (15.73–30.30)	0.1179
CRP*– mg/L	21.3 (9.4–38.4)	34.4 (16.43–50.7)	11.20 (7.125–29.58)	**0.0122**
**CCL2 (MCP-1)**–ng/mL	73.10 (20.96–169.9)	87.94 (34.74–139.1)	61.24 (22.99–143.6)	0.9334
**CCL5 (RANTES)–**μg/mL	25266 (15961–80253)	36712 (25412–83213)	24982 (14214–50209)	0.3163
**CXCL9 (MIG)**–ng/mL	2185 (489.2–11644)	4623 (825.9–9926)	2120 (368.1–6854)	0.4382
**CXCL10**– pg/mL	70.5 (25.3–448.9)	183.3 (30.03–393.5)	77.55 (25.17–273.9)	0.5736
**Fibrinogen**–mg/dL	400.2 (273.5–496.5)	437.7 (348.5–530.8)	309.5 (218.1–478.1)	0.1063
**AST**–U/L	160.2 (87.4–648.0)	268.4 (160.4–339.8)	166.3 (68.97–428.4)	0.6018
**ALT**–U/L	184.3 (132.3–498.7)	278.8 (175.1–342.9)	177.6 (112.6–352.2)	0.3946
**Total Bilirubin**–mg/dL	1.30 (0.95–1.90)	2.15 (1.20–3.175)	0.910 (0.687–1.725)	**0.0250**
Elevated Total Bilirubin**– n **(%)**	32 (38.55)	4 (66.66)	25 (27.78)	0.0755
**Elevated Total Bilirubin**–mg/dL	2.40 (1.80–3.15)	2.45 (2.03–4.53)	3.00 (2.25–3.60)	0.3305
Direct Bilirubin-mg/dL**– mg/dL	0.5 (0.3–0.8)	1.1 (0.325–1.675)	0.400 (0.200–0.700)	**0.0133**
**Indirect Bilirubin-mg/dL**–mg/dL	0.7 (0.52–1.3)	1.1 (0.8–1.5)	0.625 (0.400–1.125)	0.1366
**Elevated Indirect Bilirubin**–n **(%)**	23 (26.74)	1 (16.66)	19 (21.11)	0.5449

Data represents interquartile range, except for count/frequency of patients with Elevated Indirect or Total bilirubin, shown in percentage for each respective column. Continuous variables were compared using the Kruskal-Wallis test, while frequency data were compared using the exact Chi-Square test. Subjects were classified as having elevated creatinine levels if serum creatinine values were above 1.24mg/dL for women, and 1.29mg/dL for men; elevated total or indirect bilirubin levels were considered if values were higher than 1.5mg/dL or 1.2mg/dL respectively AU = arbitrary unit.

* = significant differences between normal creatinine subpopulation and nonsurvivors subgroup (Mann-Whitney *U* test).

** = significant differences between nonsurvivors and survivors subgroups (Mann-Whitney *U* test). AU = arbitrary unit.

**Fig 3:**
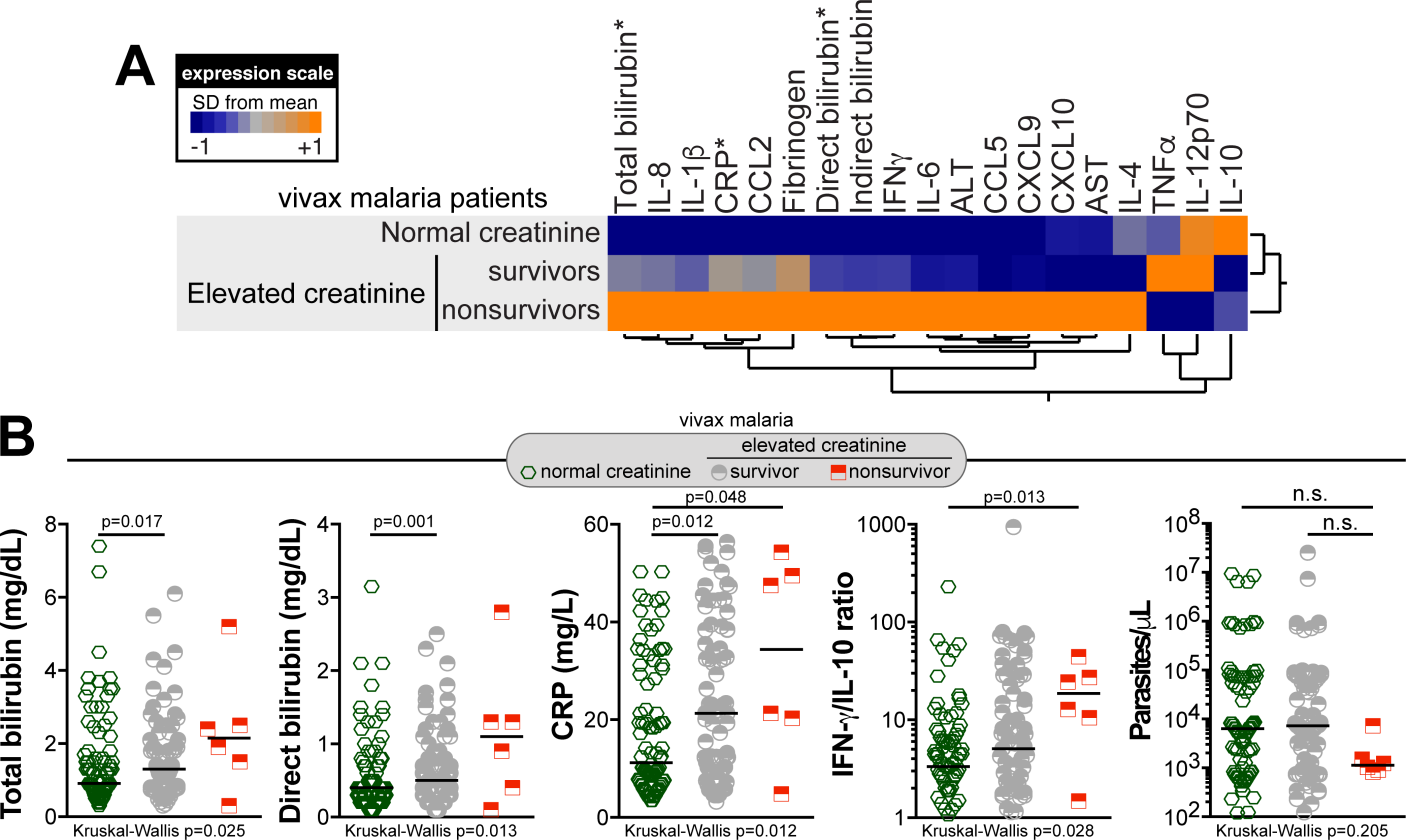
Inflammatory profile of patients with acute *Plasmodium vivax* malaria according to serum creatinine levels and outcome. (A) Overall profile of plasma concentrations of several biochemical parameters, cytokines and chemokines in patients presenting creatinine levels within the normal range (n = 90) as well as in vivax malaria survivors (n = 83) and nonsurvivors (n = 6) presenting abnormally elevated creatinine levels. Data were processed using hierarchical cluster analysis (Ward’s method) with 100X bootstrap. Dendograms represent hierarchical distance. Asterisks indicate parameters which were statistically different between the groups of malaria patients with normal or elevated creatinine levels assessed using the Kruskall-Wallis test. Scatter plots of these parameters are shown in (B). The IFN-γ/IL-10 ratio, which has been shown to correlate with the inflammatory imbalance in vivax malaria (4), and parasitemia levels were also compared between the study groups. Bars represent median values.

Hierarchical cluster analysis of circulating inflammatory markers from vivax malaria patients considering mortality and creatinine levels showed a distinct biosignature among nonsurvivors when the group is assessed separately (Fig 3A). Among the biomarkers examined, statistically significant differences between the study groups were observed in values of total and direct bilirubin, CRP and IFNγ/IL-10 ratios (Fig 3B, Table 3). Noteworthy, parasitemia levels could not distinguish these groups, considering either abnormal creatinine elevation or mortality (Table 1; Fig 3B).

## Discussion

Malaria and its complications have been extensively studied. However, kidney dysfunction related to *P*. *vivax* infections has not been completely explored. Some studies have reported cases of severe vivax malaria associated with AKI and described some of its classical clinical symptoms [10–13], whilst other studies have already depicted the immune response profile in severe vivax cases [5,6,9,26,28]. The present study adds on the current knowledge as it now addresses the associations of serum creatinine concentrations with a distinct inflammatory profile which hallmarks subpopulations at higher risk of mortality linked to *P*. *vivax* infection. This profile was especially highlighted by a strong signature composed by IFN-γ/IL-10 ratio values and CRP levels, measurements which are directly related with the degree of systemic inflammation over anti-inflammatory mechanisms. Intense systemic inflammatory responses, although non-specific, have been reportedly associated with settlement of different forms of AKI [31–33]. Our findings depict in more detail the relationships between inflammation and AKI in vivax malaria and generate hypotheses to be tested in future pathogenesis studies, rather than propose screening for cytokines in point-of-care settings.

In the study population, malaria severity was highly associated with abnormal creatinine increases. Indeed, almost 85% of the patients with severe disease presentation and more than 90% of the hospitalized patients exhibited elevated creatinine levels (Table 1). Whilst these results highlight the link between severe vivax malaria and kidney dysfunction, it is also worth highlighting that elevations in creatinine levels were not always reflected in increased disease severity, as multiple patients exhibiting elevated serum creatinine presented with nonsevere vivax malaria. The inflammatory profile observed in the group of nonsurvivors, among those with the highest levels of serum creatinine, was even more intense and unbalanced than that in the groups of malaria patients who survived with or without serum creatinine elevation (Fig 3B). However, it remains unknown whether *P*. *vivax* genetic diversity [34] would be responsible for different outcomes in those groups, with more virulent strains causing severe disease presentations. Interestingly, parasitemia levels, previously considered as a factor associated with AKI in vivax malaria patients [12,13], were not associated with increases in creatinine levels in the present study (Table 1, Fig 3B). In addition, values of CCL2 and CCL5, chemokines, which are known to be produced by kidney tubular cells during injury [31], were found increased only in the group of nonsurvivors (Fig 3A). Despite the non-significant differences in the levels of these inflammatory chemokines, it is possible that the readouts are indicating altered homeostasis in nephron’s vessels. Hence, the overall results presented here corroborate with the idea that disease severity in vivax malaria, especially considering kidney involvement, is more of a case of inflammatory imbalance and parasite-host interactions through immune activity [5,9] than heavy parasitism alone.

One of the factors that can influence serum creatinine levels is hemolysis [35], which hallmarks malaria. Although total bilirubin levels were significantly higher in patients with elevated creatinine values when compared with those with normal creatinine levels, there was no significant difference in indirect bilirubin values between those groups. Moreover, subjects with abnormally high indirect bilirubin values were most frequently not part of the group of individuals with elevated serum creatinine levels (Table 2). The opposite tendency should have been expected if mainly hemolysis was responsible for the elevation in serum creatinine levels. Hence, these results dissociate the relationship between malaria-associated hemolysis and serum creatinine elevation.

The vivax malaria patients who did not survive presented with the highest levels of creatinine in the study population, as well as with elevation of total bilirubin concentrations more dependent on the direct fraction. These results suggest an association between kidney injury and some degree of liver abnormalities and cholestasis, in line with previous reports [12–14]. Furthermore, these results could also suggest and reinforce the idea that elevations in total bilirubin levels may reflect in decreased kidney function, as hyperbilirubinemia would be associated with prerenal failure by altered water balance [36,37] and ATN [37,38]. Hence, elevated bilirubin levels may play a part in the settlement of acute kidney injury in severe vivax malaria, but probably not as the main cause, similarly to the one previously suggested in *P*. *falciparum* infections [38]. With regard to liver damage, hepatic aminotransferase values exhibited wide distribution in both groups. Additional studies are warranted to better define the relationships between kidney and liver function in severe vivax malaria.

This study presents some limitations. Information regarding the nutritional condition of the patients were not collected in the original cohort from which this study is based on. There were no follow up data on eventual dialysis or other procedures that may have been conducted during the hospitalization of severe vivax malaria patients. However, previous reports from the same population have shown that malarial treatment improved creatinine levels and inflammation [5]. Moreover, malarial treatment and renal replacement therapy have been shown previously to improve overall patient condition in other population as well [39]. AKI severity categorization was also not included in the original cohort. The RIFLE/AKIN classifications would have helped to distinguish and stratify patients based on their risk or stage of AKI, as well [40,41]. However, as delayed access to healthcare is known as a major problem in the region [23], interfering with the estimation of baseline renal function, these classifications would have little impact in the present study. Despite these limitations, serum creatinine evaluation *per se* was indeed effectively associated with worst clinical conditions and outcomes (Table 1, S1 Table, S2 Table, Fig 1). Hence, overall creatinine evaluation, bearing in mind that there are other potentially more sensitive and specific diagnostic tools [42,43], is a relevant part of physicians’ arsenal for a more efficient detection of kidney dysfunction in vivax malaria. Considering that serum creatinine assessment is widely available, its associations with worst conditions and outcomes makes it of importance for patients in certain settings.

In conclusion, the systematic analyzes of multiple inflammatory and clinical biomarkers with creatinine levels argue that renal dysfunction might be a key event in *P*. *vivax* malaria severity. Overall, disease severity was associated with elevated creatinine levels, with nonsurvivors presenting with the highest values of serum creatinine, which reinforces the hypothesis that kidney injury is highly associated with mortality in vivax malaria. The detailed inflammatory profile was depicted for each subgroup of patients stratified per creatinine levels, revealing a distinct biosignature as well as evidencing the complexity of the mechanisms leading to disease severity. Our investigation demonstrated an association between systemic inflammation and kidney dysfunction, but not an exclusive effect. We predict that the systemic inflammation observed in more severely ill malaria patients could be contributing to kidney dysfunction through different mechanisms. In addition, the overall evaluation of biomarkers suggest that liver abnormalities and hyperbilirubinemia could also play a role in severe vivax malaria-associated kidney dysfunction. The identification of key factors driving the pathogenesis of this type of disease presentation and experimental models still are necessary to guide future studies and approaches.

## Supporting information

S1 Table Baseline characteristics of the study participants. Frequency data were compared using the exact Fisher’s test or the chi-square test. Continuous variables were compared using the Kruskal-Wallis test or the Mann-Whitney *U* test. CRP, C-reactive protein; ALT, alanine aminotransferase; AST, aspartate aminotransferase. *From 165 subjects recruited as endemic controls, only 152 had all the epidemiological data available. ** From 161 subjects with non-severe vivax malaria for the variable, 160 could recall this information. ^*a*^ *=* Differences were significant between the three groups. ^*b*^ = Differences were significant between the two groups of vivax malaria patients.
(PDF)

S2 Table Clinical characteristics of vivax malaria patients. Data were compared using the exact Fisher’s test. Subjects were classified as having elevated creatinine levels if serum creatinine values were above 1.24mg/dL for women, and 1.29mg/dL for men. ^*a*^ *=* Differences were significant between the groups. * Data from only 88 patients were available. ** Data from only 87 patients were available. ^**#**^ Data from only 89 patients were available.
(PDF)

S3 Table Biochemical evaluation of healthy controls and patients with elevated and normal creatinine levels. (PDF)

S1 File STROBE checklist. (DOCX)
