# Supplementary material for: Distinct inflammatory profile underlies pathological increases in creatinine levels associated with Plasmodium vivax malaria clinical severity
Source: PLoS Negl Trop Dis. 2018 Mar 29;12(3):e0006306. doi: 10.1371/journal.pntd.0006306 (PMC5875744; doi:10.1371/journal.pntd.0006306)
Supplement: S1 Table — Frequency data were compared using the exact Fisher’s test or the chi-square test. Continuous variables were compared using the Kruskal-Wallis test or the Mann-Whitney U test. CRP, C-reactive protein; ALT, alanine aminotransferase; AST, aspartate aminotransferase. *From 165 subjects recruited as endemic controls, only 152 had all the epidemiological data available. ** From 161 subjects with non-severe vivax malaria for the variable, 160 could recall this information. a = Differences were significant between the three groups. b = Differences were significant between the two groups of vivax malaria patients. (PDF) [file pntd.0006306.s001.pdf]

S1 Table: Characteristics of the study participants

Variables	Endemic Controls	*P. vivax* malaria	
		non-severe vivax malaria	severe vivax malaria
**N**	165*	161	18
**Women**– no. **(%)**	80 (52.63)	72 (44.72)	7 (38.89)
**Age**– yrs. [a]			
Median	39	33	19.5
Interquartile interval	25-51	27-47	14.25-57.5
****Previous malaria episodes** [a,b]			
Median	13	6	1
Interquartile interval	10-18	1-12.75	0-5.25
**Years resident in the area –** no. **(%)** [a,b]			
<2yrs.	39 (25.66)	44 (27.33)	8 (44.45)
3-10yrs.	16 (10.52)	38 (23.60)	8 (44.45)
>10yrs.	97 (63.82)	79 (49.07)	2 (11.10)
**Parasitemia (count/µL)**			
Median	0	6523	3403
Interquartile interval	0	1140-72532	1001-7560
**Serum creatinine (mg/dL)** [a,b]			
Median	1.23	1.25	1.6
Interquartile interval	1.09-1.3	1.16-1.35	1.28-2.35
**CRP (ng/dL)** [a]			
Median	4.8	15.5	16.2
Interquartile interval	3.7-6.7	8.15-32.65	8.53-45.25
**AST (U/L)** [a]			
Median	42.1	165.6	180.9
Interquartile interval	33.6-54.3	85.5-642.8	58.65-259.2
**ALT (U/L)** [a]			
Median	38.9	177.1	199.5
Interquartile interval	33.2-45.75	123.7-495.2	116.4-293.2
**Total Bilirubin (mg/dL)** [a,b]			
Median	0.7	1.15	1.85
Interquartile interval	0.49-1.0	0.77-1.8	1.5-2.53
**Direct Bilirubin (mg/dL)** [a]			
Median	0.26	0.4	0.55
Interquartile interval	0.15-0.40	0.3-0.78	0.3-1.08
**Indirect Bilirubin (mg/dL)** [a,b]			
Median	0.4	0.68	1.25
Interquartile interval	0.28-0.62	0.44-1.15	1.0-1.75

Frequency data were compared using the exact Fisher's test or the chi-square test. Continuous variables were compared using the Kruskal-Wallis test or the Mann-Whitney U test.

CRP, C-reactive protein; ALT, alanine aminotransferase; AST, aspartate aminotransferase.

*From 165 subjects recruited as endemic controls, only 152 had all the epidemiological data available.

** From 161 subjects with non-severe vivax malaria for the variable, 160 could recall this information.

[a] = Differences were significant between the three groups.

[b] = Differences were significant between the two groups of vivax malaria patients.
