# Supplementary material for: Distinct inflammatory profile underlies pathological increases in creatinine levels associated with Plasmodium vivax malaria clinical severity
Source: PLoS Negl Trop Dis. 2018 Mar 29;12(3):e0006306. doi: 10.1371/journal.pntd.0006306 (PMC5875744; doi:10.1371/journal.pntd.0006306)
Supplement: S2 Table — Data were compared using the exact Fisher’s test. Subjects were classified as having elevated creatinine levels if serum creatinine values were above 1.24mg/dL for women, and 1.29mg/dL for men. a = Differences were significant between the groups. * Data from only 88 patients were available. ** Data from only 87 patients were available. # Data from only 89 patients were available. (PDF) [file pntd.0006306.s002.pdf]

S2 Table: Clinical characteristics of vivax malaria patients

Characteristic – no. (%)	Elevated Creatinine Levels	Normal Creatinine Levels	P-value
**N**	89	90	
**Fever**	89 (100)	90 (100)	1.0000
**Sweating** [a]	53 (60.23*)	32 (35.55)	0.0242
**Tachycardia**	24 (26.97)	17 (18.88)	0.2171
**Tachypnea**	9 (10.11)	6 (6.66)	0.4330
**Vomiting**	13 (14.61)	9 (9.99)	0.3722
**Oliguria**	12 (13.48)	1 (1.11)	<0.0001
**Diarrhea** [a]	17 (19.10)	5 (5.55)	0.0063
**Dehydration**[a]	21 (23.59)	9 (9.99)	0.0168
**Abdominal Pain**[a]	10 (11.24)	2 (2.22)	0.0180
**Hepatomegaly**[a]	17 (19.10)	6 (6.66)	0.0145
**Jaundice**[a]	22 (24.72)	9 (9.99)	0.0047
**Choluria**	7 (7.86)	1 (1.11)	0.0344
**Pale or clay-colored stools**	4 (4.60**)	0 (0)	0.0563
**Headache**	53 (59.55)	47 (52.22)	0.3675
**Disorientation**[a]	19 (21.35)	3 (3.37[#])	0.0004
**Seizures**	8 (8.99)	2 (2.22)	0.0574

Data were compared using the exact Fisher's test. Subjects were classified as having elevated creatinine levels if serum creatinine values were above 1.24mg/dL for women, and 1.29mg/dL for men. Oliguria was defined as estimated urinary output less than 400mL/24 h.

[a] = Differences were significant between the groups.

* Data from only 88 patients were available.

** Data from only 87 patients were available.

[#] Data from only 89 patients were available.
