# Supplementary material for: Distinct inflammatory profile underlies pathological increases in creatinine levels associated with Plasmodium vivax malaria clinical severity
Source: PLoS Negl Trop Dis. 2018 Mar 29;12(3):e0006306. doi: 10.1371/journal.pntd.0006306 (PMC5875744; doi:10.1371/journal.pntd.0006306)
Supplement: S3 Table — (PDF) [file pntd.0006306.s003.pdf]

S3 Table. Biochemical Evaluation of Healthy Controls and Patients with Elevated and Normal Creatinine Levels.

Variables	Healthy Controls	Elevated Creatinine Levels	Normal Creatinine Levels	P-value
**N**	165	89	90	
**TNF-α**– pg/mL	0 (0-9.2)	39.40 (20.75-83.80)	33.55 (12.55-78.85)	<0.0001
**IFN-γ**– pg/mL	32.0 (11.2-62.15)	132.00 (35.00-333.50)	85.48 (40.15-321.5)	<0.0001
**IL-1β**– pg/mL	5.95 (3.75-17.5)	11.40 (5.925-29.40)	10.09 (5.575-21.80)	<0.0001
**IL-4**– pg/mL	25.31 (13.49-40.31)	28.47 (18.37-113.30)	31.25 (16.16-97.15)	0.0188
**IL-6**– pg/mL	8.3 (5.2-20.2)	69.20 (22.15-140.40)	59.10 (29.13-104.10)	<0.0001
**IL-8**– pg/mL	6.03 (4.765-9.9)	32.50 (6.14-190.0)	20.54 (5.183-69.63)	<0.0001
**IL-10**– pg/mL	12.0 (7.0-20.4)	9.69 (6.43-36.83)	19.70 (6.50-59.54)	0.0469
**IFN-γ/IL-10**– AU	2.664 (0.706-5.306)	5.720 (1.675-25.21)	3.334 (1.181-6.558)	0.0001
**IL-12p70**– pg/mL	7.34 (4.865-15.05)	20.94 (10.20-33.35)	20.45 (15.73-30.30)	<0.0001
**CRP**– ng/mL	4.8 (3.7-6.7)	21.30 (9.45-38.45)	11.20 (7.125-29.58)	<0.0001
**CCL2 (MCP-1)**– ng/mL	101.2 (41.19-182.0)	73.10 (21.21-161.4)	61.24 (22.99-143.6)	0.0094
**CCL5 (RANTES)** – µg/mL	30315 (16429-46584)	25739 (18530-79892)	24982 (14214-50209)	0.3357
**CXCL9 (MIG)**– ng/mL	326.8 (181.4-488.6)	2386 (507-11548)	2120 (368.1-6854)	<0.0001
**CXCL10**– pg/mL	101.4 (35.36-211.1)	71.30 (26.07-445.8)	77.55 (25.17-273.9)	0.6468
**Fibrinogen**– mg/dL	215.4 (193.2-295.3)	400.2 (274.2-497.5)	309.5 (218.1-478.1)	<0.0001
**AST**–U/L	42.1 (33.6-54.3)	167.4 (87.62-642.8)	166.3 (68.97-428.4)	<0.0001
**ALT**–U/L	38.9 (33.2-45.75)	190.3 (133.7-495.2)	177.6 (112.6-352.2)	<0.0001
**Total Bilirubin**– mg/dL	0.700 (0.485-1.000)	1.300 (0.965-2.100)	0.910 (0.687-1.725)	<0.0001
**Direct Bilirubin**– mg/dL	0.260 (0.150-0.395)	0.500 (0.300-0.900)	0.400 (0.200-0.700)	<0.0001
**Indirect Bilirubin**– mg/dL	0.400 (0.280-0.610)	0.800 (0.530-1.300)	0.625 (0.400-1.125)	<0.0001
